# The link between childhood psychological maltreatment and cyberbullying perpetration attitudes among undergraduates: Testing the risk and protective factors

**DOI:** 10.1371/journal.pone.0236792

**Published:** 2020-09-03

**Authors:** Xiaohua Sun, Liang Chen, Yandong Wang, Yan Li

**Affiliations:** 1 Organization Department of Party Committee, University of Science and Technology Liaoning, Anshan, Liaoning Province, China; 2 School of Marxism, University of Science and Technology Liaoning, Anshan, Liaoning Province, China; 3 School of Business Administration, University of Science and Technology Liaoning, Anshan, Liaoning Province, China; 4 Student Psychological Development Guidance Center, Tsinghua University, Beijing, China; University of Connecticut, UNITED STATES

## Abstract

Based on Attachment Theory, the Barlett and Gentile Cyberbullying Model and General Aggression Model, the present study explored the relationship between childhood psychological maltreatment and cyberbullying perpetration attitudes among undergraduates, as well as the mediating roles of empathy and immorality. Using a stratified cluster random sampling method, 626 college students were tested. Structural equation modeling was used for multiple mediation analysis. Results: (1) The positive rate of childhood psychological maltreatment, referring to sustained and repeated experiencing at least one kind of psychological maltreatment, was reported by accounted for 33.87% of participants; (2) a significant positive correlation existed between childhood psychological maltreatment, immorality, and cyberbullying perpetration attitudes. However, these variables were negatively correlated with empathy (cognitive and affective empathy); and (3) there were three mediating paths: childhood psychological maltreatment was linked to cyberbullying perpetration attitudes of male college students through the mediating roles of cognitive empathy and immorality and the chain-mediating role of cognitive empathy and immorality. Conclusions: Greater experience of childhood psychological maltreatment predicted more favorable attitudes toward cyberbullying perpetration among male college students, mediated by cognitive empathy and immorality. These findings may assist parents and educators by providing effective intervention for cyberbullying perpetration attitudes.

## 1. Introduction

With the rapid development of information and communication technology, the Internet has become an important part of people’s studies, work, and lives. The 41st Statistical Report on China’s Internet Development was released by the China Internet Network Information Center in January 2018. According to the report, as of December 2017, the number of Internet users in China had reached 772 million, the Internet popularization rate was 55.8%, and the student population among all netizens was the largest at approximately 25.4%. With the popularity of the Internet and the lower age of users, cyberbullying perpetration has become a serious problem that disturbs teenagers and young adults. Cyberbullying perpetration is an act of repeatedly inflicting harm on individuals or groups with weak self-protection through electronic media [1].

Cyberbullying perpetration has cross-cultural universality. Research by Zhou in Hong Kong deduced that cyberbullying perpetration was experienced by 34.84% of students in high school and was affected by 56.88% of students [2]. Studies have corroborated that the cyberbullying perpetration of adolescents in secondary school continued when they went to university, and their style of cyberbullying perpetration showed a strong consistency [3]. Beran et al. surveyed college students in the United States and Canada and contended that 33.6% of the participants suffered from cyberbullying perpetration [4]. Survey by Zhu et al. among Chinese college students found a rate of cyberbullying perpetration of 17.32% and that cyberbullying perpetration had been experienced by 36.27% of college students, where cyberbullying participation by males was higher than that by females [5]. However, some studies have found no gender difference in the cyberbullying participation rate among teenagers [3], which may reflect cyberbullying definitions and measurement tools. Nevertheless, the anonymity, timeliness, and rapid dissemination of cyberbullying perpetration can cause serious harm to the mental and psychological status of cyberbullying victims, resulting in low self-esteem, depression, anxiety, substance abuse, and even suicide. Cyberbullying victims are likely to become new cyberbullying perpetrators, creating a vicious circle.

Scholars of cyberbullying have confirmed that attitudes and behavior are linked [6–9]. According to the Barlett and Gentile Cyberbullying Model (BGCM), perpetrators often have more positive attitudes toward cyberbullying. Studies of college students have supported this [7, 8]. BGCM posits that more accepting attitudes toward the anonymity and strength imbalance belief predicted more positive attitudes toward cyberbullying perpetration, which in turn predicted cyberbullying perpetration. Heirman and Walrave [10] found that attitudes toward cyberbullying were the strongest predictor of cyberbullying intention. Moreover, higher cyberbullying intention predicted more frequent perpetration of cyberbullying behaviors. Other studies also confirmed that cyberbullying perpetration attitudes had a significant positive predictive effect on the occurrence and frequency of cyberbullying perpetration [11, 12]. The theory of reasoned action (TRA) also suggests that attitudes, perceived behavioral control, and social norms predict intentions, which then lead to behaviors. TRA posits that one’s attitude toward a behavior and subjective norms of the behavior influence behavioral intentions, which in turn influence behavior [9]. Therefore, cyberbullying perpetration attitudes could be a stronger predictor of cyberbullying perpetration than other influencing factors. Attempts to prevent cyberbullying would do well to promote less accepting attitudes toward cyberbullying perpetration and toward perpetrators [8].

In addition, a previous study found that over 30% of college student respondents indicated that their first experience with cyberbullying was in college [13]. Moreover, individuals with a supervisory position were more exposed to cyberbullying than individuals with no managerial responsibility [6]. From a practical point of view, college students are more likely than other similar-aged people to gain a better position after graduation. Therefore, the present study aimed to explore the effects of the risk and protective factors on the mechanism of cyberbullying perpetration attitudes among undergraduates.

### 1.1 Childhood psychological maltreatment and cyberbullying perpetration attitudes

The General Aggression Model (GAM) focuses on factors associated with the individual and the situations linked with cyberbullying [6, 13]. Individual factors include personality traits, motives, attitudes, beliefs, gender, values, behavioral scripts, long-term goals, and any other consistent traits the person brings to the situation. Situational factors, conversely, are characteristics of the environment (e.g., provocation, aggressive cues, sources of frustration, external sanctions) and the degree to which the social situation restricts or offers an opportunity to act aggressively.

Previous studies have placed great emphasis on the impact of family environment on cyberbullying perpetration [14]. Studies have asserted that childhood psychological maltreatment is an important environmental predictor of cyberbullying perpetration among college students [15]. Childhood psychological maltreatment refers to the continuous and repeated adoption of a series of inappropriate behaviors by parents or other fostering parents, including terrorizing, ignoring, belittling, intermeddling, and corrupting [16]. According to attachment theory, childhood psychological maltreatment can lead to insecure-disorganized attachment, forming an early abnormal pattern and hindering the normal development of emotional regulation, impaired empathy, cognitive attribution, and stress response [17, 18], making it easy for the individual to show hostility and aggressive behavior in interpersonal communication is easy. A study by Ybarra et al. confirmed that the reported rate of poor relationships with parents among cyberbullying perpetrators was 44% and that the reported rate of good relationships with parents was 16% [19]. The correlation between childhood psychological maltreatment and cyberbullying perpetration among college students was significant. Jin et al. affirmed the significant positive correlation between childhood psychological maltreatment and cyberbullying perpetration [15].

Although some studies have suggested a childhood psychological maltreatment-cyberbullying association among college students, to our knowledge, no studies have examined the relationship between childhood psychological maltreatment and cyberbullying perpetration attitudes. Based on research on the relationship between childhood psychological maltreatment and cyberbullying perpetration, we assumed that childhood psychological maltreatment can positively predict cyberbullying perpetration attitudes among college students.

### 1.2 Empathy as a protective factor

From the perspective of the GAM, an variable likely to be correlated with cyberbullying perpetration is empathy [6, 13]. Empathy, the experience and understanding of others’ emotions based on the distinction between self and others, is both a personality trait and a psychological process [20]. Empathy is divided into two components [21, 22], namely, cognitive empathy (the ability to understand other people’s emotional emotions) and emotional empathy (the ability to experience other people’s emotional emotions). As cyberbullying does not take place face to face, cyberbullying perpetrators cannot directly observe the suffering of cyberbullying victims, thereby showing the characteristics of disinhibition and dehumanization, such as low empathy, lack of rationality, and irritability. The study elucidated that cyberbullying perpetrators had lower empathy than the control group [21, 23]. Adolescents with low levels of empathy engaged in cyberbullying perpetration at higher rates [24]. Research has shown that higher cognitive empathy contributed to decreased cyberbullying perpetration behaviors [25], especially in males [21]. Some studies have also found that in the low-level emotional empathy group, those with lower cognitive empathy experienced more cyberbullying than those with high levels. However, among women with high emotional empathy, no difference emerged in terms of cyberbullying between females with high and low cognitive empathy. Furthermore, adolescents with low affective empathy were those most likely to be cyberbullying perpetrators [23]. Nonetheless, some studies showed no effect of empathy on cyberbullying [26]. A previous study also selected TRA [27] as a theoretical framework to explain cyberbullying perpetration. The results found that the predictive effects of empathy on cyberbullying intentions and cyberbullying behaviors were mediated by attitudes, subjective norms, and perceived behavioral control [9]. However, this model did not distinguish between different subcomponents of empathy. To our knowledge, relatively few studies have researched the effect of both affective and cognitive empathy on cyberbullying perpetration attitudes [24].

According to Attachment Theory, maltreated children frequently have insecure attachments with their caregivers [28]. Research has developed a conceptual model that explores the role of psychological maltreatment in contributing to the development of impaired empathy, showing that child maltreatment was significantly negatively correlated with impaired empathy [25]. Notably, maltreated children tend to have difficulty caring for, sharing with, or taking the perspectives of others. Therefore, this study further assumes that empathy plays a mediating role in the relationship between childhood psychological maltreatment and cyberbullying perpetration attitudes.

### 1.3 Immorality as a risk factor

Although existing research has examined the causes of cyberbullying perpetration, it has rarely been systematically studied from a personality perspective. From the perspective of the GAM, the personality factor is one of the internal factors influencing an individual’s cyberbullying perpetration [13]. As part of the moral dimension of personality, immorality is the overall organization of moral cognition, emotion, and behavior formed by individuals in the process of socialization and the unity of an individual’s inner quality and mode of external moral behavior [29]. Immorality includes five components: unrighteousness, utilitarianism, indulgence, deceit, and aggression [29]. Immorality reflects the negative moral quality of personality [29]. A previous study corroborated that immorality, as part of moral personality, can negatively predict moral behavior [29]. Some empirical research has found that negative personality traits have increased aggressive behavior. For instance, autophilia, impulsive personality traits correlated with adolescent aggressive behavior, and callous-unemotional traits can predict the occurrence of cyberbullying perpetration [12]. To our knowledge, however, no previous studies have directly examined the relationship between immorality and cyberbullying perpetration attitudes.

Research has shown that childhood psychological maltreatment is significantly correlated with neuroticism [30] and impulse [31]. The researchers also investigated the relationship between family environment and college students’ immorality. The results indicated that parental passive fostering was significantly positively correlated with immorality [29]. Another study also confirmed that psychological maltreatment can result in distortions in moral engagement and identity, which in turn may lead to higher cyberbullying perpetration [32]. Furthermore, a recent empirical study suggested that self-report personality disorders may partially mediate the relationship between childhood psychological maltreatment and higher cyberbullying perpetration among university students [33]. In view of the fact that cyberbullying perpetration attitudes had a significant positive predictive effect on the occurrence and frequency of cyberbullying [12], this study assumes that immorality plays a mediating role in the relationship between childhood psychological maltreatment and cyberbullying perpetration attitudes.

### 1.4 Empathy and immorality as multistage mediating factors

The relationship between childhood psychological maltreatment and cyberbullying perpetration may be indirect. However, only a few studies have focused on the potential mediators between childhood psychological maltreatment and cyberbullying perpetration [15, 32]. According to the GAM, we proposed that empathy and immorality might play multistage mediating roles in the relationship between childhood psychological maltreatment and cyberbullying perpetration attitudes. Biehroff et al. summed up previous studies and verified that empathy was one of the important factors that constitute personality [34]. Indeed, a lack of empathy has been considered one of the essential dark personality subcomponents [35]. Studies have shown a negative link between empathy and dark personality (Machiavellianism, psychopathy, and narcissism) [35–37]. Previous studies have revealed that empathy-related constructs are linked to moral reasoning, which were also shown to be connected to personal values [38, 39]. Meanwhile, empathy and personal values predicted different types of moral schemata differently [38]. In addition, empathy not only closely correlates with people’s moral emotions and altruistic behaviors, but also plays a very important role in personality development [40]. Therefore, empathy is thought to play a mediating role preceding immortality in the hypothesized model.

In sum, although previous studies have affirmed a positive relationship between childhood psychological maltreatment and cyberbullying perpetration, few have discussed its mechanism, and the role of risk and protective factors in the mechanisms of cyberbullying perpetration attitudes have rarely been examined. Therefore, based on the GAM and attachment theory, this study examines the impact of childhood psychological maltreatment on the perpetration attitudes of college students and the mediating role of empathy and immorality.

## 2. Methods

### 2.1 Participants

The study protocol was approved by the Research Ethics Committee of the University of Science and Technology Liaoning (China). The students had to complete an informed consent form to participate in the study. Joint classes from three universities in Beijing and Liaoning Province were randomly selected from grades one to four and were tested as a group through a stratification-cluster random sampling method. A total of 700 questionnaires were distributed and 665 were returned, of which 626 were valid, resulting in an effective questionnaire rate of 94.13%. The age of the subjects ranged from 17 to 23 years, with an average age of 20.39 (*SD* = 1.46). The sample comprised 369 males (58.9%) and 257 females (41.1%). The numbers of students in engineering, science, and liberal arts were 341 (54.5%), 114 (18.2%), and 171 (27.3%), respectively.

### 2.2 Measures

#### 2.2.1 The Children’s Psychological Maltreatment Scale

The Children’s Psychological Maltreatment Scale (CPMS), compiled by Pan et al. [16], has a total of 23 items scored on a 5-point Likert scale ranging from 0 = *No* to 4 = *Always/Yes*, including five subscales: terrorizing (including 7 items, e.g., “My parents shouted at me”), ignoring (including 6 items, e.g., “My parents did not answer my questions”), belittling (including 4 items, e.g., “My parents insulted me”), intermeddling (including 4 items, e.g., “My parents read in my diary”), and corrupting (including 2 items, e.g., “My parents did not forbid me to drink”). Participants were asked to consider events that happened during childhood. A higher total score indicates a greater level of psychological maltreatment experienced by the participants. In the present study, the total Cronbach’s *α* of the scale was .92, and the values of the five subscales’ Cronbach’s *α* were .877 (terrorizing), .785 (ignoring), .860 (belittling), .746 (intermeddling), and .524 (corrupting). Although Cronbach’s *α* for the corrupting subscale is .524, it was still accepted because it contained only two items.

#### 2.2.2 The Undergraduate Immorality Adjective Evaluation Questionnaire

The UMPAEQ compiled by Wang was used [29], including 72 terms that mainly investigate four dimensions of undergraduates’ morality: immorality, kindness, selflessness, and honesty–thriftiness, among which immorality is a negative morality trait, and the others are positive morality traits. In this study, we used the subscales of immorality, which include 36 items scored on a 5-point Likert scale ranging from 1 = *strongly disagree* to 5 = *strongly disagree*. Immorality comprises five subscales: unrighteousness (including 8 items, e.g., insidious), utilitarianism (including 8 items, e.g., mercenary), indulgence (including 9 items, e.g., licentious), deceit (including 6 items, e.g., scheming), and aggression (including 5 items, e.g., despotic). The Cronbach’s *α* values of the five subscales were .782 (unrighteousness), .826 (utilitarian), .814 (indulgence), .780 (deceit), .710 (aggression), and the Cronbach’s *α* for immorality as a whole was .938.

#### 2.2.3 Basic Empathy Scale (BES)

The BES, compiled by Darrick [22] and revised by Li and colleagues [41], includes a total of 20 items along the two subscales of emotional empathy and cognitive empathy. We adopted the Emotional Empathy Scale, which contains 11 items (e.g., “I get caught up in other people’s feelings easily”), and the Cognitive Empathy Scale, which contains 9 items (e.g., “I have trouble figuring out when my friends are happy”), all scored on a 5-point Likert scale (1 = *completely disagree*; 5 = *completely agree*). Cronbach’s *α* of the emotional empathy and cognitive empathy subscale were .724 and .747, respectively.

#### 2.2.4 The Chinese version of the Cyberbullying Attitudes Measure (CAM-C)

The CAM compiled by Barlett was used [42]. The present study first obtained Barlett’s authorization for revision of the scale and then revised the CAM-C according to the standards of the International Test Commission. For the revision, exploratory factor analysis showed that the factor load of the first project was .569, and that project was retained. The CAM-C includes two subscales: Harmful Cyberbullying Attitudes (HCA; e.g., “Teasing or making fun of others with harmful comments online is fun to me”) and General Cyberbullying Attitudes (GCA; e.g., “Sending mean electronic messages to others is less harmful than face-to-face communication”), each with five items scored on a 5-point Likert scale (1 = *Completely disagree*, 5 = *Completely agree*), where a higher score indicates stronger cyberbullying perpetration attitudes. In the present study, the Cronbach’s *α* values of the HCA and GCA subscales were .747 and .882, respectively. The goodness of fit indices of the confirmatory factor analysis were *χ*^*2*^ = 248.336***, *df* = 34, CFI = .924, TLI = .899, SRMR = .049, RMSEA = .100, indicating that the construct validity of the questionnaire was acceptable.

### 2.3 Data analysis

**Fig 1:**
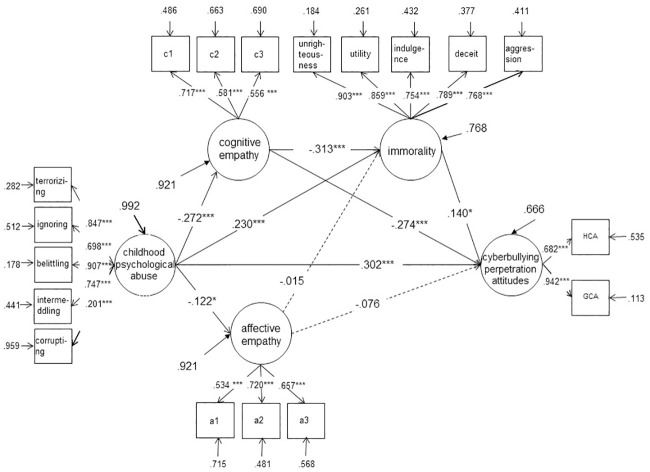
Cognitive empathy and immorality are the mediating variables between childhood psychological maltreatment and cyberbullying perpetration attitudes. Gender was entered as correlated exogenous variables to predict all other variables in the model.

SPSS23.0 was used for descriptive statistics, reliability analysis, and correlation analysis between variables. Mplus8.1 was used to analyze the confirmatory factors of each questionnaire and mediation through structural equation modeling. Multiple imputation was used in SPSS in case of missing values. As BOOTSTRAP and BCBOOTSTRAP confidence intervals of the structural equation model cannot be calculated with multiple imputation in Mplus, full formation maximum likelihood (FIML) was used to deal with missing data. Considering the large number of items in the empathy subscales, this study parceled the items of the empathy subscales according to the item parceling strategies to simplify the structure of the model [43]. First, a single-dimensional confirmatory factor analysis was conducted on each empathy subscale. The items were then parceled to ensure that the average score of the normalized factor loadings in each item parceling was nearly equal. Therefore, each empathy subscale was divided into 3 item parcels (a1, a2, a3 for affective empathy and c1, c2, c3 for cognitive empathy) as the observation index (see Fig 1).

## 3. Results

### 3.1 Common method bias test

Harman’s single factor test method was used to conduct exploratory factor analysis on the data to avoid common method biases in the analysis resulting from collecting data by the self-evaluation method. If a factor extracted or a certain factor explained by one of several factors is relatively large and if the first factor has an explanatory power higher than 40%, then common method bias exists [44]. The results confirmed that a total of 28 factors with characteristic roots greater than 1 were extracted, and the first factor explanatory power was only 16.64%, lower than the criterion of 40%. Therefore, common method bias was not found using this method in the present study.

### 3.2 Correlation analysis

A total of 212 participants (33.87%) reported a childhood psychological maltreatment rate greater than/equal to 1, and within that subsample, 36 students (5.75%) reported a maltreatment rate greater than/equal to 2. According to the definition of positive rate of childhood psychological maltreatment in previous studies, an average score ≥ 1 was the examination standard, indicating that the participants showed sustained and repeated experiences of at least one kind of psychological maltreatment [16, 31]. If the average score was ≥ 2, the participants had experienced a greater intensity of childhood psychological maltreatment than others.

Correlation analysis results confirmed that a significant correlation existed between childhood psychological maltreatment, empathy, immorality, and cyberbullying attitudes (Table 1). Among them, a significant positive correlation existed between childhood psychological maltreatment, immorality, and cyberbullying perpetration attitudes (*p* < .01). A significant negative correlation existed between empathy (cognitive and affective empathy) and childhood psychological maltreatment, immorality, and cyberbullying perpetration attitudes (*p*s < .01). A gender difference test showed that the childhood psychological maltreatment, immorality, and cyberbullying perpetration attitudes scores were significantly higher in male participants than in females, while the affective empathy score of females was significantly higher than that of males.

**Table 1:** Correlation matrices and gender differences in key variables (*N* = 626).

Variables	*M*	*SD*	% of Missing	*t*	1	2	3	4	5
1 CPM	.85	.56	.45	3.67***	1				
2 affective empathy	3.50	.52	.26	−5.02***	−.20**	1			
3 cognitive empathy	3.73	.49	.32	−1.30	−.21**	.26**	1		
4 immorality	1.81	.56	.29	5.59***	.30**	−.17**	−.28**	1	
5 CPA	1.88	.66	.11	4.77***	.36**	−.17**	−.30**	.33**	1

**p < .01,

***p < .001,

CPM: childhood psychological maltreatment; CPA: cyberbullying perpetration attitudes.

### 3.3 Analysis of mediation

This study used a structural equation model with a bootstrapping and Markov chain Monte Carlo (MCMC) method to test the mediating effects of empathy and immorality, controlling for gender as a correlated exogenous variable. First, the significance of the total effect c was examined. In this study, the total effect of childhood psychological maltreatment on cyberbullying perpetration attitudes of college students was 0.434 (c), and the total effect coefficient was significant (*p* < .001). All fit indices of the total effect model were acceptable (*χ*^*2*^ = 102.853***, *df* = 18, CFI = .954, TLI = .929, SRMR = .045, RMSEA = .087). We then tested the models split by gender. The total effect was 0.417 (c) for boys and 0.453 for girls, and the total effect coefficients were significant (*p*s < .001). The goodness of fit indices of the total effect model for boys (*χ*^*2*^ = 36.942***, *df* = 13, CFI = .976, TLI = .962, SRMR = .034, RMSEA = .071) were all acceptable. Although RMSEA was .110, the other goodness of fit indices of the total effect model for girls (*χ*^*2*^ = 53.428***, *df* = 13, CFI = .949, TLI = .918, SRMR = .045) were less than the critical value.

**Fig 2:**
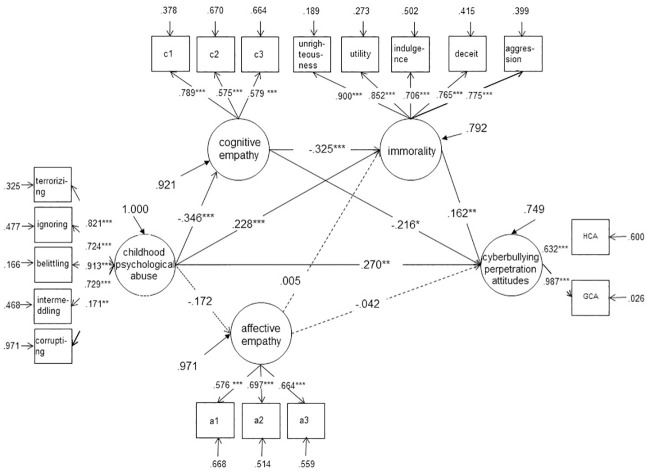
Cognitive empathy and immorality as mediating variables between childhood psychological maltreatment and cyberbullying perpetration attitudes in boys.

**Fig 3:**
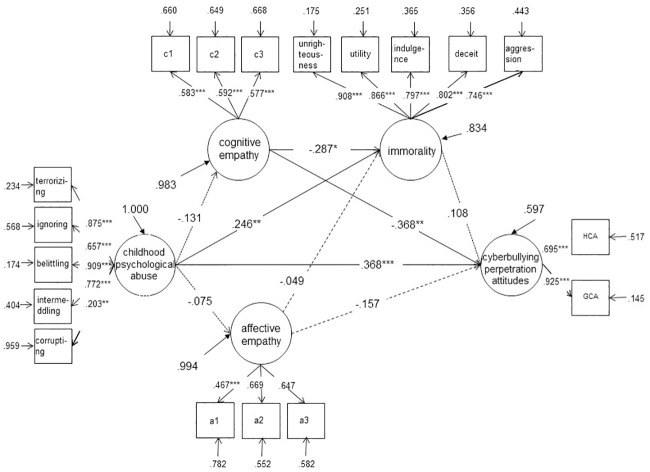
Cognitive empathy and immorality as mediating variables between childhood psychological maltreatment and cyberbullying perpetration attitudes in girls.

Second, the significance of the path coefficients was examined. Based on the hypotheses, model A was constructed (see Fig 1). Structural equation modeling analysis showed that the path coefficients of affective empathy → immorality (*r* = −.015, *p* > .05) and affective empathy → cyberbullying perpetration attitudes (*r* = −.076, *p* > .05) were not significant. However, the analysis indicated that the goodness of fit indices of the model were acceptable (*χ*^*2*^ = 547.335***, *df* = 139, CFI = .917, TLI = .897, SRMR = .067, RMSEA = .069), and the other path coefficients and normalized factor loadings of the observational variables reached significance, indicating that the model was acceptable. Similar results were found for boys, as the path coefficients of childhood psychological maltreatment → affective empathy (*r* = −.072, *p* > .05), affective empathy → immorality (*r* = −.005, *p* > .05), and affective empathy → cyberbullying perpetration attitudes (*r* = −.042, *p* > .05) were not significant (see Fig 2). The goodness of fit indices of the model for boys were acceptable (*χ*^*2*^ = 341.813***, *df* = 126, CFI = .921, TLI = .904, SRMR = .072, RMSEA = .068). However, no mediating effects were found for the girls’ group (see Fig 3). Given that childhood psychological maltreatment had a significant direct predictive effect on the cyberbullying perpetration attitudes of college students, cognitive empathy and immorality played a part in mediating the linkage of childhood psychological maltreatment to cyberbullying perpetration attitudes in college boys.

Moreover, the confidence interval of the path coefficients was estimated for boys by bootstrapping with 1000 repetitions (see Table 2). The resulting 95% confidence interval for the total indirect effect of empathy and immorality did not contain 0 (.036, .246), indicating that the two mediator variables had significant mediating effects between childhood psychological maltreatment and cyberbullying perpetration attitudes of college students (.130, accounting for 31.15% of the total effect). The mediating effect included four important indirect effects: First, the confidence interval of indirect effect 1 via the path of childhood psychological maltreatment → cognitive empathy → cyberbullying perpetration attitudes did not contain 0 (.023, .147), indicating that cognitive empathy has a significant mediating effect between childhood psychological maltreatment and the cyberbullying perpetration attitudes of college students (.075, accounting for 17.92% of the total effect). Second, the confidence interval of indirect effect 3 via the path of childhood psychological maltreatment → immorality → cyberbullying perpetration attitudes did not contain 0 (.008, .065), indicating that the mediating effect (.037, accounting for 8.86% of the total effect) produced by this path also reached significance. Third, the confidence interval of indirect effect 4 via the path of childhood psychological maltreatment → cognitive empathy → immorality → cyberbullying perpetration attitudes did not contain 0 (.005, .034), indicating that the mediating effect (.018, accounting for 4.37% of the total effect) produced by this path reached significance.

**Table 2:** Bootstrap analysis of the mediating effect test for boys.

Influence path	Standardized indirect effect estimation	95% confidence interval	
		lower limit	upper limit
CPM → cognitive empathy → CPA	(−.346) × (−.216) = .075	.023	.147
CPM → immorality → CPA	.228 × .162 = .037	.008	.065
CPM → cognitive empathy → immorality → CPA	(−.346) × (−.325) × .162 = .018	.005	.034

CPM: childhood psychological maltreatment; CPA: cyberbullying perpetration attitudes.

## 4. Discussion

This study examined the relationship between childhood psychological maltreatment and cyberbullying perpetration attitudes in college students. First, we found that childhood psychological maltreatment accounted for 33.87%, which is basically the same as the research results (30.19%) [15]. Hence, it is fairly common in a general Chinese college student population. Second, the results of the present study confirmed that childhood psychological maltreatment had a significant positive predictive effect on the cyberbullying perpetration attitudes of college students, and childhood psychological maltreatment was found to be an important risk factor for cyberbullying perpetration attitudes among college students. This finding was consistent with a previous study, in which, after controlling for the effects of physical maltreatment and neglect, Holmes confirmed that childhood psychological maltreatment can significantly predict adolescents’ aggressive attitudes [45]. According to attachment theory, childhood psychological maltreatment undermines the safe attachment relationship between children and dependents, forming a negative “internal working mode” [17, 18]. With the difficult development of social emotions, emotional disorders [46–48], such as anxiety, depression, and hostility, occurred in interpersonal communication. In addition, peer relationship problems also occurred, such as externalizing problem behavior as violent attacks. A previous study confirmed that retaliation was one of the motives for cyberbullying perpetration. Victims of school violence could retaliate through emails and text messages and vent their anger and resentment through cyberbullying perpetration [3]. Given the anonymity, convenience, and diffuseness of the network, college students with childhood psychological maltreatment were more likely to alleviate their accumulated negative moods in interpersonal conflicts through cyberbullying perpetration. Cyberbullying perpetration is less stressful and anxious than face-to-face attacks. Therefore, victims of cyberbullying are more inclined to retaliate through cyberbullying perpetration, a strategy for childhood self-defense found in the psychological maltreatment of college students. The impact of childhood psychological maltreatment on the cyberbullying perpetration attitudes of college students was verified in the present study within an Eastern Asian cultural context.

In terms of gender differences, boys’ cyberbullying perpetration attitudes scores were significantly higher than those of girls. This finding was consistent with those of some previous studies [49]. A meta-analysis by Kowalski et al. verified that males were reported to have higher attitude scores for cyberbullying perpetration than females in some studies, but others showed the opposite pattern or no gender differences [13]. A possible reason is that researchers used different cyberbullying definitions and measurement tools to examine different types of cyberbullying perpetration (such as phone bullying and email bullying). College students’ cyberbullying perpetration attitudes were examined in the present study. For instance, the first item is “If someone scolds me, the best way is to attack them online” rather than directly attacking them, and reflects the degree of recognition of cyberbullying perpetration when college students are responding to interpersonal conflicts. Gilligan [50] asserted that males seek fairness in moral judgment, where females have a tendency to solicitude. As an aggressive behavior, cyberbullying perpetration causes serious injury to interpersonal relationships. Therefore, when males encounter unfair situations, the degree of recognition of cyberbullying will be significantly enhanced. Conversely, females are more concerned with coordinating interpersonal relationships when making moral judgments, which reduces their acceptance of cyberbullying perpetration because of their solicitude tendency. Previous studies have confirmed this view. Yang and Wang [51] affirmed that higher moral judgment ability hardly reduces the positive influence of moral disengagement on male aggression, but it can significantly reduce the positive impact of moral disengagement on female aggression.

Apart from investigating the relationship between childhood psychological maltreatment and the cyberbullying perpetration attitudes of college students, this study also examined the mechanisms between the two. The results showed childhood psychological maltreatment to be associated with the cyberbullying perpetration attitudes of college students through the mediating roles of cognitive empathy and immorality in boys, and the effects of such roles are exerted through three paths: separate mediation of cognitive empathy and immorality, and chained mediation of cognitive empathy-immorality. This result is partly consistent with the basic view of the GAM, where aggression is the result of the interaction between the external environment and internal factors. In interaction with the external environment, individuals internalize them into an automated “internal working model” to guide behavior. According to attachment theory, empathy originates from children’s safe attachment experience [17, 52]. Parents with strong empathy can encourage children to explore their parents’ ideas, thereby promoting the development of children’s social intelligence and forming good interpersonal relationships [52].

Contrariwise, childhood psychological maltreatment can lead to the formation of insecure attachment, which undermines the development of empathy and internalizes negative work patterns. Children believe that they are imperfect, unlovable, hostile, or dismissed by others, and that interpersonal interactions are potentially risky and unpleasant. This negative working mode enables children to easily feel contempt, fear, and distrust when interacting with others, thereby hindering the development of empathy. Given the limited development of empathy, children who suffer childhood psychological maltreatment are commonly self-centered in interpersonal communication, paying more attention to their own feelings and needs than to the feelings and opinions of others. In the long run, it is easy for them to develop immorality, such as abandonment, utilitarianism, and indulgence. Biehroff et al. contend that empathy is an important factor constituting personality [53]. However, it should be noted that the predictive effects of affective empathy on immorality and cyberbullying were not significant, even though there were significant correlations between these variables, indicating that affective empathy did not play a mediating role. Affective empathy refers to experiencing and sharing others’ emotions. In contrast to face-to-face interactions, virtual social interactions in cyberspace are characterized by a paucity of social and contextual cues [13]. According to the Reduced Social Cues Model [54], a lack of affective feedback in the form of reduced social and contextual cues could lead to a deficiency, especially in affective empathy, as a result of deregulation of cyberbullying perpetration [55]. A previous study showed that participants with high levels of cognitive empathy and low levels of affective empathy reported less frequent cyberbullying than those with low levels of both cognitive empathy and affective empathy among Singaporean adolescents [21]. Thus, the role of cognitive empathy appears to be more important than affective empathy in predicting cyberbullying behaviors.

In addition, childhood psychological maltreatment positively correlates with immorality. Jiang and Xu [56] concluded that childhood maltreatment was significantly correlated with antisocial personality behavior. Antisocial personality patients commonly exhibit a general pattern of behavior that ignores or infringes on the rights of others, such as noncompliance with social norms, fraud, violence, and attack tendencies [56]. Therefore, individuals suffering from childhood psychological maltreatment were easily cyberbullied in the face of pressure and interpersonal conflicts due to the popularity of the Internet and the convenience of communication tools in college life.

One of the limitations of this study is probably the use of a sample that is not representative of all Chinese college students. The participants were mainly science or engineering students. Thus, the research should be extended to Chinese college humanities students to verify the results of this study in a broader population. Another limitation of this study is the use of cross-sectional data to gauge the mechanisms between variables over time. Further studies should use longitudinal data to clarify the causality of the relationships and illuminate the underlying mechanism. In addition, common method biases are inevitable in survey data for several reasons. Since the data used in the current study were collected from self-reported questionnaires, it is possible that participants underreported their participation in cyberbullying. Therefore, other research methods should also be considered in future studies. For example, it would be interesting to use an interview method to test the effects of parenting styles and cyberbullying attitudes on the mechanisms of cyberbullying in future studies. A third limitation of this study is the lack of cyberbullying perpetration in the hypothesis model. Although cyberbullying perpetration attitudes are an important predictive variable for cyberbullying perpetration, it would be better to construct a model that includes cyberbullying perpetration and influential factors in the future.

The present study also confirmed that in the relationship between childhood psychological maltreatment and college students’ cyberbullying perpetration attitudes, the mediating effect of cognitive empathy was the largest, reaching 14.06%, indicating that college students’ cyberbullying attitudes were mainly linked through the intermediary path of cognitive empathy from childhood psychological maltreatment. Cognitive empathy was found in this study to be the key protective factor that reduced college students’ cyberbullying perpetration attitudes. In the future, an intervention study of college students’ cyberbullying perpetration can be carried out through cognitive empathy training. For example, college students could be asked to view the real consequences of cyberbullying (e.g., complaints from the cyberbullying victim’s perspective), and trained to recognize the cyberbullying victim’s emotions, rather than engaging in typical responses of cyberbullying victim blaming [57]. Based on our results, other potentially effective intervention methods include providing adolescents with cyberbullying intervention programs, improving parent–child relationships [21], and training parents to increase their awareness of cyberbullying.
